# Impacts of invading alien plant species on water flows at stand and catchment scales

**DOI:** 10.1093/aobpla/plv043

**Published:** 2015-04-30

**Authors:** D. C. Le Maitre, M. B. Gush, S. Dzikiti

**Affiliations:** 1 CSIR Natural Resources and the Environment, PO Box 320, Stellenbosch 7599, South Africa; 2 Centre for Invasion Biology, Department of Botany and Zoology, University of Stellenbosch, Private Bag X1, Matieland 7602, South Africa

**Keywords:** Hydrological impacts, invasive alien plants, vegetation structure, water resources, water-use

## Abstract

There have been many studies of the diverse impacts of invasions by alien plants but few have assessed impacts on water resources. We reviewed the information on the impacts of invasions on surface runoff and groundwater resources at stand to catchment scales and covering a full annual cycle. Most of the research is South African so the emphasis is on South Africa's major invaders with data from commercial forest plantations where relevant. Catchment studies worldwide have shown that changes in vegetation structure and the physiology of the dominant plant species result in changes in surface runoff and groundwater discharge, whether they involve native or alien plant species. Where there is little change in vegetation structure [e.g. leaf area (index), height, rooting depth and seasonality] the effects of invasions generally are small or undetectable. In South Africa, the most important woody invaders typically are taller and deeper rooted than the native species. The impacts of changes in evaporation (and thus runoff) in dryland settings are constrained by water availability to the plants and, thus, by rainfall. Where the dryland invaders are evergreen and the native vegetation (grass) is seasonal, the increases can reach 300–400 mm/year. Where the native vegetation is evergreen (shrublands) the increases are ∼200–300 mm/year. Where water availability is greater (riparian settings or shallow water tables), invading tree water-use can reach 1.5–2.0 times that of the same species in a dryland setting. So, riparian invasions have a much greater impact per unit area invaded than dryland invasions. The available data are scattered and incomplete, and there are many gaps and issues that must be addressed before a thorough understanding of the impacts at the site scale can be gained and used in extrapolating to watershed scales, and in converting changes in flows to water supply system yields.

## Abstract (precis)

The impacts of invasions by alien plant species on the quantity of rainwater that reaches rivers and streams have been studied in several countries. Some studies have found that there is a large impact and others have found little or no impact. These conflicting conclusions can be explained largely by differences in the structure (e.g. height, depth of root systems) and the physiology (e.g. evergreen, deciduous, water stress tolerance) between the alien and the indigenous plant species. The greater the differences between the two, the greater the impact is likely to be.

## Introduction

There is a growing body of knowledge on the biophysical and socio-economic impacts of terrestrial invasions by alien (introduced) plant species (Levine *et al*. 2003; Ehrenfeld 2010; Pyšek and Richardson 2010; Vilà *et al*. 2011; Pyšek *et al*. 2012; Rejmánek and Richardson 2013; Dickie *et al*. 2014; Funk *et al*. 2014). However, the hydrological impacts of these invasions at a stand or catchment scale—such as the impacts on surface runoff, groundwater recharge and evaporation losses—have received comparatively little attention. Exceptions include studies on the impacts of riparian invasions in the western USA (Doody *et al*. 2011; Hultine and Bush 2011), *Salix* invasions in Australia (Doody and Benyon 2011; Doody *et al*. 2014) and various species in South Africa. Although this review emphasizes research done in South Africa, we show that the findings are globally relevant because there are general hydrological principles that determine the direction and magnitude of the impacts as discussed by Le Maitre (2004). This paper explores those principles in greater detail and addresses concerns about the quantitative basis and generalizability of research on the hydrological impacts of invasions raised by Hulme *et al*. (2013).

Research on impacts of invasions in South Africa has had a strong hydrological emphasis largely because of the country's climate, lack of forests and historical background. It is a dry country which receives less than half the global mean annual rainfall [±490 mm/year (hydrologists often express water quantities in millimetres depth, the same as the unit for rainfall; to convert to volumes—1 mm/year is equivalent to 10 m^3^/ha/year)] and has <0.5 % of its area under forest (Mucina and Rutherford 2006). Therefore, it requires plantations of alien tree species to meet its needs for timber and fibre (Richardson *et al*. 2003; van Wilgen and Richardson 2014). Systematic planting of alien tree species for timber production began in the late 1800s but, by the 1930s, growing concerns about the hydrological impacts of these plantations led to a government-funded research programme (Kruger and Bennett 2013). This research demonstrated that afforestation results in substantial decreases in river flows in the affected catchments (Bosch and von Gadow 1990; Dye 1996*a*; Scott and Smith 1997). The prominence of alien tree species as invaders raised concerns that their impacts on water flows could be similar (Wicht 1945; Kruger 1977). When renewed concerns about these impacts were raised in the 1990s (Le Maitre *et al*. 1996), the lack of data on the invasion impacts resulted in the use of the afforestation research data for estimating the impacts of alien tree invasions in South Africa on water resources, particularly the effect on river flows (Le Maitre *et al*. 1996, 2000; Calder and Dye 2001; Cullis *et al*. 2007; van Wilgen *et al*. 2008). The preliminary results of this research were a key motivation for the establishment of the government-funded Working for Water programme (van Wilgen *et al*. 1998) and have been used in setting priorities for investments in control measures so that resources are effectively deployed (Forsyth *et al*. 2012). The state of knowledge of the hydrological impacts of invasive alien plants in South Africa was last reviewed by Görgens and van Wilgen (2004). There have been several additional studies since 2004 that have advanced the understanding of invasive alien plant water-use (e.g. transpiration and interception losses) and how this affects hydrological processes and river flows.

We first review the current understanding of the key factors that limit the water-use of plants at stand and catchment scales to establish a basis for understanding the impacts of invading species. We then summarize and place the information on hydrological impacts into the context of those limiting factors and draw some generalizations. We finally discuss some fundamental challenges for research. All the catchment-level studies of the impacts of invasive species in South Africa have been based on managed plantations of these species rather than invasions, so information on the impacts of plantations has also been included. The emphasis of this review is on stand and catchment scale impacts because a thorough review of studies at the leaf, plant, stand and catchment scale, and the coupling and decoupling between the different levels is given by Asbjornsen *et al*. (2011) and for invading versus native species by Cavaleri and Sack (2010). We have also focussed on data that covers at least one year as that time frame is meaningful for informing management decisions such as prioritizing control.

## Key Limiting Factors

Hydrologists have been constructing and refining conceptual approaches for understanding and predicting the relationships between rainfall, surface runoff, evaporation and vegetation at a range of scales for more than a century (Budyko 1974; Eagleson 1978; Reich *et al*. 1997; Rodriguez-Iturbe *et al*. 1999; Andréassian 2004; Cavaleri and Sack 2010; Asbjornsen *et al*. 2011; Moore and Heilman 2011; Moles *et al*. 2012; Van Bodegom *et al*. 2012; Porporato and Rodriguez-Iturbe 2013). Calder (2005) proposed a relatively simple and practical way of understanding the key factors controlling evaporation. It involves four sets of factors: three are physical and one involves plant traits. He also proposed that no more than two can be the primary controls in a given situation. The physical factors are: (i) energy availability from solar radiation or, in certain situations, advected energy; (ii) soil moisture availability, especially in strongly seasonal climates; and (iii) precipitation droplet size and its effect on interception. The plant traits are: (i) plant physiology, including whether it is evergreen or deciduous and its moisture stress tolerance; and (ii) plant size above ground (height, stem diameter, leaf area) and depth of the root system. Calder's limits concept therefore links hydrology and plant traits to explain how vegetation plays a critical role in regulating water fluxes in the terrestrial component of the water cycle. The principles proposed by Moore and Heilman (2011) are similar but include more detail on root systems and soil characteristics.

### Climatic factors

Evaporation is the second largest component of the hydrological cycle after rainfall, so an understanding of the controls on evaporation is critical. The widely used and emulated model by Budyko (1974) for global and regional estimates of evaporation and, by deduction, surface runoff and other measures of liquid water fluxes, takes an important step in this direction. It is based on two fundamental relationships:

$$\begin{array}{rl} & \text{Long - term climate dryness index}(ø)\\ & \;=\frac{\text{potential evaporation}(E_{p})}{\text{precipitation}(P)}\\ & \text{Long - term evaporative index}(\epsilon_{0})\\ & \;=\frac{\text{actual evaporation}(E_{a})}{\text{precipitation}(P)} \end{array}$$

These relationships are derived from the basic principle that actual evaporation in dryland situations is limited at the arid end of the climatic range by the supply of water (*P*) and at the humid end by the atmospheric evaporative demand (energy availability). The reason for the long-term measurements is that, in the short-term, inter-annual variations in rainfall, and thus soil moisture storage, and exploitation of soil moisture by deep-rooted plant species introduced to environments dominated by shallow-rooted species, can result in evaporation exceeding annual rainfall (Dye 1996*b*; Scott and Lesch 1997; Jarmain and Everson 2002; Farley *et al*. 2005; Clulow *et al*. 2011; Mendham *et al*. 2011). Plants growing in situations where additional water is available, such as on floodplains and over shallow aquifers, can also maintain high transpiration rates (Dye and Jarmain 2004; Engel *et al*. 2005; Farley *et al*. 2005; Doody *et al*. 2011; Moore and Heilman 2011; O'Grady *et al*. 2011). However, in terrestrial settings without supplementary sources of water, this imbalance cannot be maintained indefinitely, so the mean evaporation from vegetation will not exceed mean rainfall in dryland situations. In most cases, it will be less because of rainwater losses through evaporation from soil and litter, as well as runoff and water percolation beyond the reach of root systems to recharge groundwater.

Using data on long-term rainfall and runoff from large catchments, Budyko (1974) found that the evaporative index can be estimated from the long-term climate dryness index using a curvilinear function *ε*_b_ = (*θ* tanh *θ*^−1^(1 − cosh *θ* + sinh *θ*))^0.5^ with an error of ∼10 % [(Donohue *et al*. 2010), Fig. 1]. This model shows that (in large catchments) the evaporative index generally does not approach either the energy limit or the water limit (Donohue *et al*. 2010; Zhang and Chiew 2012). These two limits to evaporation apply to dryland situations or large catchments when environments with additional water amount to a small fraction of the total area: the water limit because long-term actual evaporation cannot exceed long-term rainfall; and the energy limit because long-term actual evaporation cannot exceed the energy available to drive it over these time spans.

**Figure 1.**
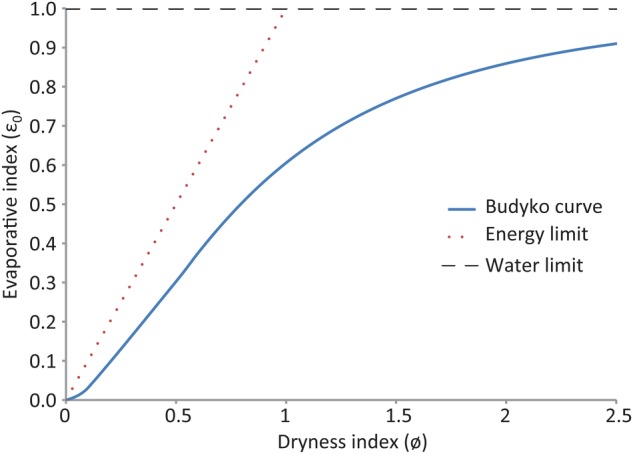
The Budyko curve for the relationship between the long-term dryness index and the evaporative index and the energy and water limits to long-term evaporation.

The energy limit can also be exceeded in some situations where additional energy is available to drive evaporation, for example via advection (Everson 1999; Calder 2005). This is often the case for wetlands, oases or riparian woodlands in landscapes where prevailing winds introduce warm, dry air and increase evaporative demand (Everson 1999; Calder 2005). The relationships also predict that as rainfall increases, evaporation becomes a decreasing proportion of the rainfall as the energy limitation decreases both potential and actual evaporation (Fig. 1) (Zhang *et al*. 2001).

**Figure 2.**
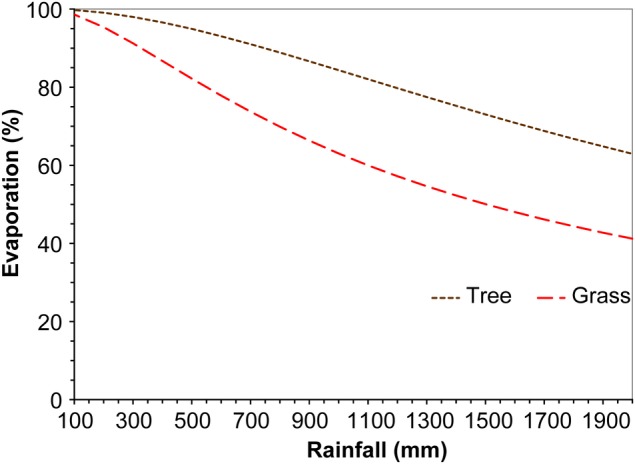
Generalized relationships between mean annual rainfall and mean annual evaporation (as a percentage) for catchments with two differing dominant vegetation types: grasslands, mainly seasonal pastures or trees (woodlands or forest). The evaporation data represent dryland settings as riparian zones comprise a small proportion of most catchments (1–5 % and up to 10 % in some cases) or had vegetation which did not change when the rest of the catchment was converted from tree to grass cover. Derived from data in Zhang *et al*. (1999, 2001).

There are deviations from the Budyko curve which are primarily due to two kinds of factors: (i) climatic—air temperatures which are directly related to evaporative demand (Thornthwaite 1948; Komatsu *et al*. 2012) and rainfall seasonality (Potter *et al*. 2005; Zhang *et al*. 2008); and (ii) vegetation—structure, eco-physiology and deciduousness (Bosch and Hewlett 1982; Scott *et al*. 2004; Brown *et al*. 2005; Calder 2005; Donohue *et al*. 2010, 2012). Zhang *et al*. (1999, 2001) analysed the effect of vegetation properties on the relationships between mean annual rainfall and mean annual evaporation for a dataset of more than 300 catchments. They derived general relationships between evaporation and rainfall for catchments either under: (i) grasslands (seasonal pastures or herbaceous vegetation) or (ii) evergreen woodland or forest (Fig. 2). Other studies have confirmed that incorporation of vegetation features can improve the accuracy of such models (Donohue *et al*. 2010; Komatsu *et al*. 2012) in line with Calder's (2005) proposals on limiting factors. Research to date clearly shows that increases in woody plant density or replacement of grasslands by woody plants almost invariably increases the evaporation and decreases water availability by reducing surface runoff and groundwater recharge (Huxman *et al*. 2005; Wilcox and Thurow 2006).

### Water availability

In dryland settings, water availability is limited by the proportion of rainfall captured and stored within the soils and underlying weathered material (regolith). However, there are situations where additional water is available from or via groundwater within the rooting zone such as in alluvial (riparian) or colluvial deposits or in deep soils and weathered profiles. Measurements in riparian invasions (Dye and Jarmain 2004), or after clearing riparian trees in natural settings, plantations or invaded catchments (Dye and Poulter 1993; Prinsloo and Scott 1999; Scott 1999; Everson *et al*. 2007; Salemi *et al*. 2012), show that water-use by the same species in the riparian zone is higher than in adjacent dryland situations. However, these studies also show that there is substantial spatial and temporal variability, both along and across the flood plain (Engel *et al*. 2005; Scott *et al*. 2006; Hultine and Bush 2011; Salemi *et al*. 2012). Some of this is due to vegetation characteristics but much of it is due to variations in the accessibility and volume of the additional water caused by, for example, variations in the depth to the water table and heterogeneities in the water storage capacity and transmissivity of the soils and the aquifer material (Scott *et al*. 2008; O'Grady *et al*. 2011; Funk 2013). In addition, the native vegetation in these habitats may have a similar structure and water-use characteristics offsetting the gains from clearing and resulting in little or no net (incremental) change in water-use (Scott 1999; Doody *et al*. 2011). Pertinent examples are the high evaporation rates reported for native riparian vegetation and riparian invasions by *Acacia mearnsii* (Dye and Jarmain 2004) or *Salix babylonica* invasions and native riparian eucalypt forest (Doody *et al*. 2011), or where deep soil moisture or groundwater is being exploited (Jobbágy and Jackson 2004; Engel *et al*. 2005; Farley *et al*. 2005; Benyon *et al*. 2006; Cleverly *et al*. 2006; Fritzsche *et al*. 2006; Kagawa *et al*. 2009; Clulow *et al*. 2011).

### Plant traits

Vegetation structure and deciduousness affect interception and transpiration. Plant size was used by Le Maitre *et al*. (1996) as a key determinant of invasive plant water-use. This was logical given the wide range of growth forms of invaders and the lack of data on the impacts on streamflow except for commercial plantation species (pines, eucalypts) and how they compared with native grass, woodlands and fynbos shrublands. However, the relationship between total biomass and the biomass of the leaves (and thus the transpiring and intercepting leaf area) varies between growth forms and over the lifespan of a plant, especially in trees. Although these changes follow allometric rules that also relate to plant water-use (Enquist *et al*. 1999; West *et al*. 1999; Niklas *et al*. 2003; Poorter *et al*. 2012; Zeppel 2013), they indicate that biomass *per se* is not a reliable indicator of transpiration (or interception).

At the plant level, a number of plant traits play key roles in regulating and limiting evaporation (i.e. both transpiration and interception losses) (Lavorel *et al*. 1997; Lavorel and Garnier 2002; Calder 2005; Moore and Heilman 2011; Van Bodegom *et al*. 2012). Key traits relating to water-use can be divided into two related groups: (i) the plant size (e.g. height, leaf area, root system depth); and (ii) physiology [e.g. hydraulic architecture (cavitation resistance, water flux rates, stomatal control), evergreeness]. One of the key relationships in woody plant species is the area of the sapwood and the leaves, which are related through what is known as the Huber value—the ratio of sapwood to leaf area (Carter and White 2009). The Huber value quantifies the ability of the plant xylem to conduct water and maintain transpiration. High ratios tend to occur in species which are conservative water users and tolerant of moisture stress, and low ratios in species which are not moisture stress tolerant (Tyree and Sperry 1988; McDowell *et al*. 2002). Unfortunately, data on sapwood areas and Huber values are available only for woody species with typical secondary growth, and so exclude many important invading species. A more widely available measure is the leaf-area index (LAI, m^2^ of leaf/m^2^ of ground within the canopy or at stand scale) which is a particularly important ecophysiological parameter because it is related to both the ability of the plant to absorb energy and to transpire water or intercept rainwater (Larcher 1975). So, it is directly linked to plant water-use and growth rate or productivity. Combined with leaf longevity or evergreeness and stomatal conductance, it provides a direct link to plant water-use and is, therefore, a potentially very useful factor for explaining why invasions by, or plantations of, certain species have more significant impacts on water resources than others (Le Maitre 2004; Everson *et al*. 2007, 2011; Clulow *et al*. 2011; Fink and Wilson 2011).

Plants are also known to vary in their transpiration per unit leaf area (WU/LA). Thus there are species with higher WU/LA but a lower LAI than other species, and even variations within a species (Hatton *et al*. 1998; Carter and White 2009). These complications can be circumvented by the use of micro-meteorological and remote-sensing techniques for estimating evaporation at the canopy and stand scale via the energy balance. But they still require data about the canopy structure and the canopy or surface conductance at the sampling sites. Satellite-based remote sensing, as used by Meijninger and Jarmain (2014) and Jarmain and Meijninger (2012), can provide a basis for scaling-up micro-meteorological and stand-level measurements to landscape and catchment scales, but more work is needed before this approach can be applied across the rugged landscapes typical of high water yielding mountain catchments and for mixtures of species.

Nevertheless, the strong relationships involving LAI explain why it and other leaf-related indices of vegetation vigour or productivity (e.g. specific leaf area) are widely used in remote-sensing-based assessments of vegetation productivity and evaporation (Bastiaanssen *et al*. 1998; Gower *et al*. 1999; Asner *et al*. 2003; Cleugh *et al*. 2007; Glenn *et al*. 2011; Mu *et al*. 2011; Velpuri *et al*. 2013) and more widely in modelling vegetation dynamics (Running and Coughlan 1988; Landsberg and Waring 1997; Wright *et al*. 2001; Woodward and Lomas 2004; Dovey 2005). Other leaf traits also associated with competitiveness may account for greater water-use by invading species but the distinctions are not always clear cut (Grotkopp *et al*. 2002; Grotkopp and Rejmánek 2007; Tecco *et al*. 2010; Drenovsky *et al*. 2012). Other comparisons among invasive species have found that they have, among others, higher leaf nutrient levels and specific leaf area and lower wood densities (Diaz *et al*. 2004; Leishman *et al*. 2007; Cavaleri and Sack 2010; Ordonez *et al*. 2010; Funk 2013; Tecco *et al*. 2013).

Major invading plant species whose stand and landscape-level water-use have been documented include a wide range of growth forms, height, evergreeness and root depth (Table 1). Most of them are evergreen trees or shrubs, with the trees roughly divisible into the two groups used by Le Maitre *et al*. (1996), namely tall trees and medium trees. Deep root systems are also common among the trees with some taxa, such as *Eucalyptus* and *Prosopis* being well known for having root systems that reach depths of 10–20 m or more (Canadell *et al*. 1996; Schenk and Jackson 2002*a*, *b*; Stromberg 2013). The effective depths of root systems are often misunderstood because the majority of the root mass is concentrated in the upper 0.5 m of the soil (Jackson *et al*. 1996). However, deep-rooted species often are characterized by having a few roots, sometimes called sinker roots, which can reach great depths and are specialized for water transport (Pate *et al*. 1995; Dawson and Pate 1996; McElrone *et al*. 2004; Stromberg 2013) and may utilize hydraulic lift and redistribution (Oliveira *et al*. 2005). Deep root systems have been reported for pines in deep sands in Zululand, South Africa (Haigh 1966), and also for Eucalyptus in settings where the soils are considered shallow but there is deep weathering (Dye 1996*b*). It is likely that invading taxa with this trait will also be exploiting deep soil moisture or groundwater provided it is accessible.

**Table 1.** A summary of information on key traits which are known to affect transpiration and interception rates of invading plant taxa including plantations of these species. Information on typical plant height was taken from Henderson (2001) or based on personal observations (^#^). Root depths from Canadell *et al*. (1996) and Schenk and Jackson (2002*a*, *b*) with deep roots reaching >2 m depth. Asterisk indicates LAI calculated from data in the source. Type of LAI estimate: C, individual canopy based; S, stand based.

Taxon	Growth form	Height (m)	Evergreen	Deep roots (m where known)	LAI	Sources for LAI data and notes
*Acacia mearnsii*, *A. dealbata*, *A. decurrens* (Wattles)	Tree	>5	Yes	>4	2.0–3.5; 3.5; 2.3	S: Dye and Jarmain (2004); Everson *et al*. (2007) after canopy closure; Bulcock and Jewitt (2010)—4 years old; Bulcock and Jewitt (2012)—5 years old
*Acacia saligna*	Tree	<10	Yes	Probably	Moderate	S: Morris *et al*. (2011)—NDVI 0.63 versus native 0.51 which indicates greater growth and water-use potential
*Chromolaena odorata*	Scrambler	<4^#^	Yes	Unlikely	2.5–5.6	C: Slaats *et al*. (1996)
*Eucalyptus* spp.	Tree	>8	Yes	>10	3–4 at 4 years old, 1.7 at 10 years; 1.2–5.3 (4.25); 1.2–4.5; 2.6; 2.7	S: Dye (1996*a*, *b*); Dye and Jarmain (2004); Dovey (2005); Engel *et al*. (2005); Mendham *et al*. (2011); Bulcock and Jewitt (2010)—10 years old; Bulcock and Jewitt (2012)—5 years old; Dye (1996*b*) reported roots up to 30 m depth
*Hakea* spp.	Shrub	<6	Yes	<4	3.81, 2.85; 4.51	C: Read *et al*. (2006) for *H. dactyloides* and *H. teretifolia* respectively; S: Moles *et al*. (2005) estimate from BIOME4 model
*Lantana camara*	Scrambler	<3^#^	In some areas	No	0.5, 1.5	S: Gush (2011)
*Pinus* spp.	Tree	>8	Yes	<8	8.8; 4.6–5.5; 2.57–2.66*; 2.9, 3.8	S: van Laar (1984); Williams *et al*. (2006); Dillon *et al*. (2001); Bulcock and Jewitt (2010)—12, 15 years old
*Populus* spp.	Tree	>8	No	<4	3.1; 4.0–7.0 (5.8); 1.8–2.8; 2.1–3.3; 2.74	S: van Laar (1983); Cleverly *et al*. (2006); Gazal *et al*. (2006) (intermittent-perennial); Nagler *et al*. (2005*a*); Glenn and Nagler (2005); Dye *et al*. (1996); Dye *et al*. (2008)
*Prosopis* spp.	Tree	<5^#^	No	>10	1.2–1.4; 1.4–3.8 (mean 2.4) tree form; 1.15–1.42 (mean 1.28) shrub form	C: Dzikiti *et al*. (2013*b*), Kiniry (1998), S: Sharifi *et al*. (1982), Nagler *et al*. (2009)
*Salix babylonica*	Tree	>5	No	<4	3.28	S: Glenn and Nagler (2005), native willow forest
*Solanum mauritianum*	Shrub	<10	Probably	No	Moderate	S: White *et al*. (2009)—measured values not available
*Tamarix chinensis*	Tree	<6	Probably	No	0.9–3.5 (3.0); 1.2–4.2; 2.6–3.6; 2.58–4.05 (3.58)	S: Nagler *et al*. (2005*a*, *b*); Cleverly *et al*. (2006); Nagler *et al*. (2009)

Leaf-area index data were available for some taxa and they show quite a range of LAI values both between and within some taxa (Table 1). Some of the values are affected by the form of the leaves and how the LAI was calculated. Needle-leaved species generally have high LAIs while values for reed-like plants such as *Arundo donax* with photosynthetic stems or leaf sheaths depend on the interpretation of the leaf form and area. Many species can achieve an LAI of more than two, implying that they have the potential for high transpiration rates and potentially high interception losses. Shrub or scrambler species like *Chromolaena* have high LAI values (Table 1) and relatively high annual evaporation rates (Table 2), which suggests that their impacts will be closer to those of trees than their growth form indicates.

**Table 2.** A summary of data on evaporation from stands dominated by invading plant species, including selected information from commercial forest plantations of those species. Mean annual rainfall has been adjusted to the mean for the catchment rather than a particular rain gauge. Remote sensing-based estimates of annual evaporation from stands with a canopy cover of at least 35 % in KwaZulu-Natal and the Western Cape in South Africa were taken from Jarmain and Meijninger (2012) and Meijninger and Jarmain (2014). K, KZN, KwaZulu-Natal; W, Western Cape; T, transpiration plus shaded water evaporation.

Taxon	Site	Method	Annual rainfall (mm)	Annual runoff (mm)	Estimated evaporation (mm/year)	Sources and notes
*Acacia mearnsii*, *A. dealbata*, *A. decurrens* (wattle species)	Working for Water sites	Remote sensing			740 ± 145^K^, 925 ± 225^W^	Meijninger and Jarmain (2014)
*Acacia mearnsii*	Seven Oaks, midlands, KZN, plantation	Bowen ratio	616–1016		1048–1364	Dye and Jarmain (2004), plantation excluded riparian zone (∼10 %)
	Two Streams, midlands, KZN, plantation	Catchment gauging	659–1170 (MAP 853)	7–46	701–1121	Clulow *et al*. (2011) mature stand October 2000–September 2004, included riparian zone
		Scintillometry	689–819		1156–1171	Clulow *et al*. (2011), 1–2 years old stand
*Acacia saligna*	Working for Water sites	Remote sensing			600 ± 195^W^	Meijninger and Jarmain (2014)
*Chromolaena odorata*	Working for Water sites	Remote sensing			1020 ± 215^K^	Meijninger and Jarmain (2014)
*Eucalyptus grandis*	Tzaneen, Limpopo, plantation	Catchment gauging	1368	209	1159	Scott *et al*. (2000*a*), mean for mature plantation
	Sabie, Mpumalanga, plantation	Catchment gauging	1155	15	1140	Scott *et al*. (2000*a*), streamflow ceased after 8 years, mean for mature plantation
*Eucalyptus grandis*	Sabie, Mpumalanga	Sap flow	1459		1347^T^	Dye *et al*. (1996)
*Eucalyptus dunnii*, *E. macarthurii*	Seven Oaks, midlands, KZN, plantation	Bowen ratio	616–1016		1246–1618	Jarmain and Everson (2002)
*Eucalyptus* spp.	Working for Water sites	Remote sensing			575 ± 195^K^, 945 ± 230^W^ (largely riparian)	Meijninger and Jarmain (2014)
*Centaurea solstitialis*	Two sites, central California	Soil moisture decrease	491 and 744		105–120 more than annual grasses	Gerlach (2004), the annual grasses also were invaders replacing perennial grasses and forbs
*Centaurea solstitialis*	Shasta valley, northern California	Soil moisture declines	460		158 perennial grass 118 annual grass 99	Enloe *et al*. (2004), annual grasses also invaders
*Hakea* spp.	Working for Water sites	Remote sensing			830 ± 240^W^	Meijninger and Jarmain (2014)
*Lantana camara*	Working for Water sites	Remote sensing			965 ± 140^K^	Meijninger and Jarmain (2014)
*Pinus caribaea*	Viti Levu, Fiji	Micro-meteorological model	1707		1926—6 years old, 1717—15 years old	Waterloo *et al*. (1999)
*Pinus patula*	Cathedral Peak, Little Berg, KZN, plantation	Catchment gauging	1531–1616	466–473	1065–1143	Scott *et al*. (2000*a*), mean for mature plantation
	Sabie, Mpumalanga, plantation	Catchment gauging	1149	13	1136	Scott *et al*. (2000*a*), streamflow ceased 16 years after planting
	Usutu, Swaziland	Heat-pulse velocity	1124		944	Dye *et al*. (2008)
*Pinus radiata*	Jonkershoek, Western Cape, plantation	Catchment gauging	1346–1416	280–408	990–1136	Scott *et al*. (2000*a*), mean for mature plantation
*Pinus radiata*	Mt Gambier, South Australia, plantation	Sap flow, ground water levels	630		540–975 no groundwater access; 1074–1344 with groundwater access	Benyon *et al*. (2006), mature plantations
*Pinus* spp.	Working for Water sites	Remote sensing			915 ± 265^W^	Meijninger and Jarmain (2014)
*Populus* spp.	Greytown, highlands KZN	Sap flow	±900		818	Dye *et al*. (2008), probably underestimated
*Prosopis* spp.	Rugseer, Kenhardt, Northern Cape	Sap flow	150	±0	25–35	Fourie *et al*. (2007); Dzikiti *et al*. (2013*b*)
*Salix babylonica*	New South Wales, Australia	Sap flow, water balance	400		Active river channels 1755–2410; 563 for river bank trees	Doody *et al*. (2011); Doody and Benyon (2011)
*Solanum mauritianum*	Working for Water sites	Remote sensing			945 ± 125	Meijninger and Jarmain (2009)

## Measurements of Invasive Species Water-use

### Dryland invasions

There is a large body of information globally on the effects of different vegetation types and changes in vegetation types on runoff and other components of the hydrological cycle. Most of it is on the effects of changes in the natural vegetation, or from natural vegetation to cultivated land, but there is information on differences between, or changes from, non-woody to woody vegetation or changes in the structure of woody vegetation (e.g. Bosch and Hewlett 1982; Zhang *et al*. 2001). Most of the studies of hydrological impacts have focused on dryland invasions and most of these were on the short-term effects on soil moisture balance or evaporation and not on runoff (Levine *et al*. 2003; Cavaleri and Sack 2010). Estimates of evaporation are also available for invasions or plantations of some of the taxa whose traits were summarized in the previous section (Table 2). The impacts of commercial forest plantations have typically been reported as streamflow reductions compared with matched control (unafforested) catchments (e.g. Scott *et al*. 2000*a*), but the reductions can be converted to evaporation using the water balance equation and matching rainfall data (Table 2) (Bosch and von Gadow 1990; Dye 1996*a*). The data show that evaporation for closed stands varies from ∼1050 to 1350 mm/year and reached 1600 mm/year for young *Eucalyptus grandis* (Dye 1996*a*). Pines can reach high evaporation rates as well in suitable climates (Table 2) (Waterloo *et al*. 1999), sometimes with very high interception rates (Calder 1991). The results of these studies cannot always be compared directly because some of the catchments were only partially afforested. When the reductions are expressed as millimetres per 10 % planted, they vary between sites based on growing conditions: 20–53 mm/year for *Pinus radiata* (warm climate, high winter rainfall, deep soils, Jonkershoek), 36–60 mm/year for *Pinus patula* (cold climate, high summer rainfall, deep fertile soils Cathedral Peak) and 48 mm/year (Sabie, warm climate, high summer rainfall, deep fertile soils), to 48–50 mm/year, for *E. grandis* (Tzaneen, similar to Sabie) (Scott *et al*. 2000*a*).

Some species may have relatively high evaporation rates although they are not trees, for example *Chromolaena* and *Lantana* (Table 2). Data for plantations of the major species were also given by Meijninger and Jarmain (2009) and Jarmain and Meijninger (2012). They were lower than those reported for catchment studies: pines in the Western Cape 735 ± 215 mm/year, eucalypts and wattles in KwaZulu-Natal were 690 ± 190 and 615 ± 140 mm/year, respectively, but still higher than the natural vegetation they had replaced. Early studies of interception in eucalyptus plantations in Mpumalanga and pines in Jonkershoek (Western Cape) found that they were typically low for plantation tree species because of the high intensity of rainfall and temporal pattern of events (<10 % of total evaporation) (Dye 1996*a*). However, more recent studies by Everson *et al*. (2007) and Bulcock and Jewitt (2012) in the mist-belt region of KwaZulu-Natal, which is characterized by low-intensity rainfall events, recorded high interception losses in *A. mearnsii* (±30 %), *E. grandis* (±15 %) and *P. patula* (±21.4 %). These differences are consistent with a limiting factor of raindrop size (Calder 2005) in addition to rainfall amount and intensity per event. More research is needed to determine how representative the high and low values are of other areas and under different rainfall intensity regimes and for a range of stand-ages, densities, leaf-area indexes and site conditions. Studies of plantation species suggest that water-use efficiency is also an important factor, with the key difference between native and introduced tree species being the slow growth rates of the native tree species rather than differences in water-use (transpiration rates) (Wise *et al*. 2011).

A couple of studies were of herbaceous weeds and found substantial changes in water-use relative to natural vegetation which are likely to result in changes in water flows. A decrease of 56 % in runoff after simulated rainfall was observed in an area of perennial grassland invaded by the thistle *Centaurea maculosa* (Lacey *et al*. 1989). *Centaurea solstitialis* invasions in annual grasslands resulted in a reduction in soil moisture equivalent to 1050–1200 m^3^/ha/year in one study (Gerlach 2004), while ET increased by 40 mm/year (23 %) compared with native perennial grasslands in another (Enloe *et al*. 2004). *Centaurea maculosa* is short-lived (<10 years) but is deep-rooted and forms multi-aged stands, whereas *C. solstitialis* is an annual but has deep roots and continues growing after the grasses have senesced; both maintain a high canopy cover (additional information from USDA NRCS 2013).

In summary, the differences in evaporation, and thus in water discharges, between native vegetation and matched invasions (or tree plantations) show that invasions typically have a higher water-use than native vegetation. The differences are consistent with expectations given the changes in vegetation structure (e.g. height, root depths, LAI) and physiology (e.g. deciduousness) (Calder 2005; Moore and Heilman 2011; Funk 2013). Thus, the hydrological impacts of invasive alien plant species are not special or exceptional, although differences in their physical and physiological traits may allow them to maintain greater water-use than the native species they replace. Information about these traits can be used to provide more robust estimates of the water-use of species whose water-use has not yet been measured.

### Riparian invasions

In riparian or floodplain settings (or areas with aquifers accessible by plant roots), theory predicts and data show that evaporation will be greater than the adjacent dryland areas because water availability is no longer the primary limiting factor (Scott 1999; Dye and Jarmain 2004; Calder 2005; Hultine and Bush 2011; Moore and Heilman 2011; Salemi *et al*. 2012). Evaporation from riparian invasions by *A. mearnsii* exceeded that for the native vegetation in the Western Cape and in the KwaZulu-Natal midlands by ∼171 and 424 mm/year, respectively (Table 3) (Dye and Jarmain 2004). The greater annual evaporation in the Western Cape was attributed largely to high daily transpiration rates during the dry, hot summer. The greater difference between invaded and natural sites in KwaZulu-Natal was primarily due to seasonal (winter) dormancy in the riparian grassland. In both cases, the evaporation in the adjacent dryland communities was lower than that for the riparian communities. *Pinus* species growing in a riparian zone were found to use ∼200 mm/year more water than pines in the adjacent dryland fynbos (Table 3) (Dzikiti *et al*. 2013*a*). Short-term increases in low flows of 9–31 m^3^/ha/day have been reported after clearing riparian invasions (Dye and Poulter 1993; Prinsloo and Scott 1999; Rowntree and Beyers 1999; Everson *et al*. 2001). The relative gains in streamflow from riparian versus dryland clearing of plantation trees range from 3.35 times at Biesievlei to ∼2.39 times at Two Streams (Table 3). These are substantial gains but they are also short-term and will decrease in the long-term as the native vegetation re-establishes itself (Scott 1999), as found in the Two Streams study (Everson *et al*. 2007; Clulow *et al*. 2011). The extent of the decrease will depend on the ability of the native species to access the same sources of additional water. Where the native species root systems are as deep as those of the invaders, there will be little or no gain in the long-term, but where they are much shallower the long-term gains could be large.

**Table 3.** Observed and modelled evaporation and impacts on streamflow for native and invaded riparian settings, including afforested riparian zones in plantations. MAP, mean annual precipitation; Et, evaporation. ^a^Calculated using the results of the break point modelling in the report.

Location	Climate	Vegetation, treatment	Results	Source
Groenberg, Wellington and Drakenstein, Paarl, Western Cape	Winter rainfall MAP^1^ ±1050 mm, ±906 mm respectively	*Acacia mearnsii* (dense)	Et^2^ 1503 mm/year	Dye and Jarmain (2004)
Jonkershoek, Stellenbosch, Western Cape	Winter rainfall MAP 1324 mm	Restioid (evergreen reed) floodplain wetland	Et 1332 mm/year	Dye and Jarmain (2004)
	Winter rainfall MAP 1200–2600 mm	Dryland, tall fynbos	Et 600–900 mm/year	Scott *et al*. (2000*a*)
Gilboa, midlands, KwaZulu-Natal	Summer rainfall MAP 867 mm	*Acacia mearnsii* (dense)	Et 1260 mm/year	Dye and Jarmain (2004)
		Riparian grassland	Et 836 mm/year	Dye and Jarmain (2004)
Midlands and Drakensberg, KwaZulu-Natal	Summer rainfall MAP 700–1500 mm	Grasslands	Et 600–860 mm/year	Schulze (1979)
Biesievlei, Stellenbosch, Western Cape	Winter rainfall MAP 1400 mm	*Pinus radiata* plantation	Et 1057 mm/year from water balance	Scott *et al*. (2000*a*)
		Clearing riparian pines	Streamflow increase 11 503 m^3^/ha/year	Scott (1999)
		Clearing dryland pines	Streamflow increase 3 430 m^3^/ha/year	Scott (1999)
Simonsberg, Stellenbosch, Western Cape	Winter rainfall MAP ±812 mm	*Pinus pinaster*, *P. halepensis*, self-sown ±20 years old, 2 years of data	Riparian 980, 1417 mm/year	Dzikiti *et al*. (2013*a*)
			Non-riparian 753, 1190 mm/year	Dzikiti *et al*. (2013*a*)
Witklip, Sabie, Mpumalanga	Summer rainfall MAP 996 mm	Grassland, 34 % pine plantation with unplanted riparian zone	Et 632 mm/year	Scott (1999)
		Clearing riparian scrub lightly invaded by pines and eucalypts	Streamflow increase 7966 m^3^/ha/year	Scott (1999)
		Clearing dryland pines	4044 m^3^/ha/year	Scott (1999)
Seven Oaks, midlands, KwaZulu-Natal	Summer rainfall MAP ±840 mm	*Acacia mearnsii* plantation	Et 1048–1364 mm/year	Jarmain and Everson (2002)
Two Streams, midlands, KwaZulu-Natal	Summer rainfall MAP 853 mm 689–819 for 2007 and 2008	*Acacia mearnsii* plantation	Et 1156–1171 mm for 2007 and 2008 MAR 2000–2008—48 mm	Clulow *et al*. (2011)
		Clearing of riparian *Acacia mearnsii*	Streamflow increase of 6.47 m^3^/ha/year^a^	Everson *et al*. (2007)
		Clearing of dryland *Acacia mearnsii*	Streamflow increase of 5.62 m^3^/ha/year^a^	Everson *et al*. (2007)
South-western USA	Summer rainfall, arid climate (<250 mm/year)	*Tamarix* species, invader	220–1500 mm/year, mean 765; 851–874 mm/year; mean 950 mm/year	Doody *et al*. (2011), Table III; Nagler *et al*. (2005*b*); Nagler *et al*. (2010)
		*Populus* spp., *Salix* spp., native	1000–1200 mm/year; 484–968 mm/year	Dahm *et al*. (2002); Scott *et al*. (2006)
New South Wales, Australia	Summer rainfall (400 mm/year *S. babylonica*; 900 mm/year *S. fragilis*)	*Salix babylonica*	1755–2410 mm/year active river channels; 563 mm/year river banks	Doody and Benyon (2011)
		*Salix fragilis*	1216–1340 mm/year	
		*Eucalyptus* spp./mixed native riparian	550–1320 mm/year	
Rugseer River, near Kenhardt, Northern Cape, South Africa	Summer rainfall, arid climate (<250 mm/year)	*Prosopis* species invasion of the floodplain alluvium of this ephemeral river	25 mm/year, groundwater fluctuation before and after clearing	Fourie *et al*. (2007)
			35 mm/year, sap flow, energy balance, groundwater levels	Dzikiti *et al*. (2013*b*)

When the Two Streams study began, the plantation was already established and there was almost no streamflow although it was the middle of the rainy season (Everson *et al*. 2007). The runoff to rainfall ratio was only 2.18 % from 1 January 2000 to 30 April 2004 but increased to 7.2 % in the period after clearfelling (December 2003–November 2008) despite re-afforestation in 2006. Little lateral water flow reached the riparian zone while the dryland trees were present although the deeper soils remained moist (Everson *et al*. 2007; Clulow *et al*. 2011). The sap flow data (larger diameter trees have greater sap flow rates), and greater diameters of riparian zone trees (1.53 times the dryland trees), provide strong evidence that transpiration was greater in riparian than non-riparian trees.

An important finding was made by the Two Streams study, namely that the evaporation from the *A. mearnsii* stand (largely dryland) exceeded the annual rainfall by a substantial margin (Table 1) (Clulow *et al*. 2011). This supported the findings of previous studies (Scott and Lesch 1997; Jarmain and Everson 2002). The ability to maintain such high transpiration rates seems to be mainly due to the trees developing deep root systems (>4.8 m deep) which exploited the moisture stored in the sub-soil and regolith (Clulow *et al*. 2011). These findings support the conclusions of other studies which have suggested that plantation trees are able to deplete soil and regolith water stores (Dye 1996*b*; Clulow *et al*. 2011). The observations also explain how afforestation of some catchments has dried up the streams completely, in some cases resulting in lags of a few years between clearing and streamflow recovery to pre-afforestation conditions (Scott and Lesch 1997; Scott *et al*. 2000*a*). Unsustainable soil moisture exploitation could also be happening at other sites where there are dryland invasions by deep-rooted species on deep soils and weathered material. Where deep-rooted trees have been present for some years, it may require some time to replenish the soil moisture storage and restore the normal water balance, in some cases more than a year (Scott and Lesch 1997; Scott 1999; Everson *et al*. 2007).

Two assessments of the water-use of riparian tree invasions in arid environments have been done in South Africa. The one estimated a groundwater loss of 50.4 m^3^/month during the growing season (October–February), or ∼251.9 m^3^/ha/year (25 mm/year) by *Prosopis* species hybrids (Fourie *et al*. 2007). However, the canopy cover of the *Prosopis* stand in that study was only ∼21 % so the equivalent for a closed plant canopy was roughly 120 mm/year. The other recorded peak transpiration by *Prosopis* species stands of ∼80 m^3^/ha/month and a total annual use of ∼345 m^3^/ha/year (35 mm) (Dzikiti *et al*. 2013*b*). The canopy cover was ∼31 % so the equivalent for a closed plant canopy would be ∼111 mm/year. These estimates assume that all this groundwater could be saved by clearing *Prosopis* but the long-term saving would depend on the water-use of the native tree species that replace the *Prosopis*. The Rugseer River, where these studies were done, is ephemeral with extended dry periods (it is only estimated to flow for ∼36 % of the time) and its catchment gets ∼150–250 mm/year of rainfall so groundwater availability is very limited. Plant moisture stress measurements showed high pre-dawn stress levels (values lower than −3.0 MPa, Dzikiti *et al*. 2013*b*), which explains the low transpiration rates.

These estimates are low compared with those for native *Prosopis* woodlands in the southern USA of 350–1100 mm/year, with most studies giving estimates between 350 and 750 mm/year (Le Maitre 1999; Scott *et al*. 2000*b*, 2004, 2006, 2008; Nagler *et al*. 2005*a*, *b*) but these were all on floodplains of perennial rivers. The evaporation from the native woody vegetation found along South African river systems in arid environments has only been briefly assessed. Everson *et al*. (2009) reported daily evaporation on a few summer days from riparian vegetation in the non-perennial Seekoei River as varying from 2.8 to 3.3 mm. This is potentially lower than for *Prosopis* invasions given that the native species seldom approach the density and canopy cover of *Prosopis* invasions (Le Maitre 1999; Wise *et al*. 2012). *Prosopis* trees also tend to produce new leaves earlier in the spring than native riparian species (Van den Berg 2010). Wise *et al*. (2012) used an estimated difference of ±600 m^3^/ha/year (60 mm/year) for dense floodplain (riparian) invasions based on the available literature. Some studies have found large reductions similar to those reports for South Africa. The indications are that *Prosopis* species are likely to use more groundwater than the equivalent native species in South Africa and in similar settings in other countries where they are invasive (e.g. India, Australia, Ethiopia, Kenya).

Although there are studies of the water-use of poplars grown for biomass, the growing conditions and silvicultural treatments (typically intensive management) are so different from those in invasions and native forests that their findings are not applicable. Two studies have examined poplar species (*Populus deltoides*) water-use in South Africa (Dye *et al*. 2008). Poplar plantations are typically located on alluvial soils but are not usually planted up to the actual river banks. The modelled annual transpiration was ∼818 mm with peak values of 6–8 mm/day during the period from October to December. The trees were very susceptible to a fungal disease which meant that transpiration began declining in early January rather than in April when leaf-shedding should begin. The annual estimate was adjusted to compensate for this to some extent but it is probably still conservative. However, studies of riparian poplar forests in the western USA provide similar estimates of evaporation (Table 3).

An assessment of the impacts of willow (*S. babylonica*) invasions on river systems in Australia found that evaporation differed substantially between trees growing in the active (flowing) river channel and trees on the banks (Doody and Benyon 2011; Doody *et al*. 2011). In active river channels, the total annual evaporation ranged from 1755 to 2410 mm/year (transpiration plus shaded water evaporation) compared with 563 mm/year for river bank invasions and open water evaporation of 1396–1604 mm/year). The mean annual rainfall in the study area was 404 mm suggesting there were markedly different moisture regimes between banks and active channels because there would be little or no lateral groundwater inflow to the floodplain. Although *S. babylonica* is deciduous, its annual evaporation rates may exceed those reported for evergreen species (e.g. Table 1) provided there is sufficient water available.

Early reports on *Tamarix* invasions in the USA estimated a daily water-use reaching 200 m^3^/ha/day (20 mm/day (Sala *et al*. 1996), resulting in an estimated total flow reduction of 1.4–3.0 billion m^3^/year for the larger rivers of the western USA (Zavaleta 2000). But more recent estimates suggest that tamarisk water-use is about the same volume of water as, or even less than, the native riparian forest species they replace (Table 1) (Scott *et al*. 2008; Nagler *et al*. 2009; Doody *et al*. 2011; Hultine and Bush 2011; Moore and Owens 2012).

The same may be true of *A. donax* invasions in California where there are native reeds and other species (Watts and Moore 2011). However, *Arundo* invasions in South Africa are often more extensive than those of *Phragmites*, and also occur in situations where *Phragmites* and *Typha* are absent (D. C. Le Maitre, pers. obs.), which could increase its impacts compared with native riparian species. *Arundo* also tends to remain evergreen while *Phragmites* and *Typha* die back in the winter which may affect the relative water-use.

In summary, studies of invasive species in riparian settings have confirmed that their annual transpiration or evaporative water-use can exceed that of the native riparian vegetation, provided that there are changes in vegetation structure, phenology or other traits. This means that the impacts of riparian invasions can be much greater than that is indicated by their extent or the proportion of the landscape that can be categorized as riparian. The differences seem to be less in environments where the native riparian vegetation is evergreen (e.g. fynbos) than where the native vegetation is deciduous (e.g. grasslands, savanna) and the invaders are evergreen (Dye and Jarmain 2004).

## Challenges for Research

### The variety of species

**Figure 3.**
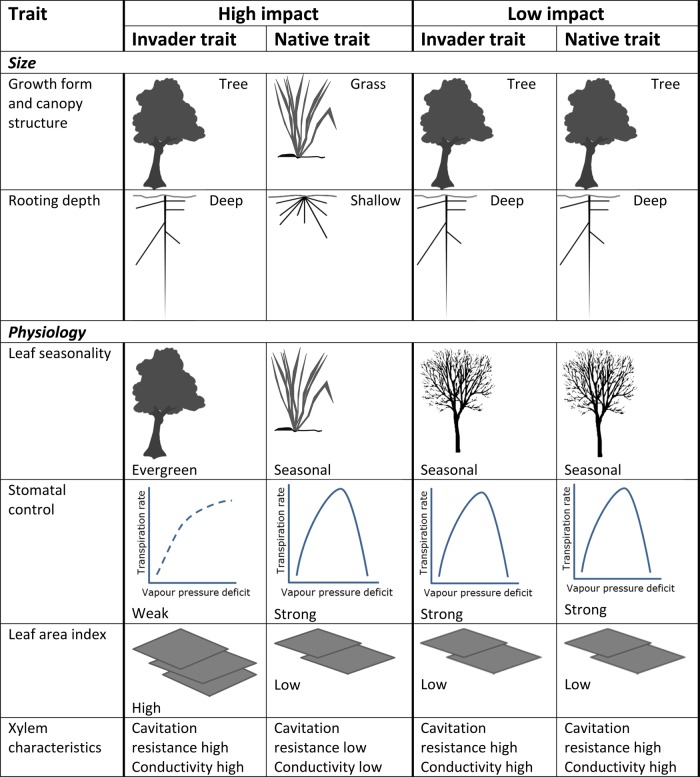
Effects of combinations of major plant traits that have been found to influence the impacts of plant invasions on water resources relative to natural vegetation (after Calder 1991, 2005; Le Maitre 2004). Plant traits are inter-related but can be grouped into those related to size and those related to physiology. High impacts on water resources will occur where there are marked contrasts in these traits (e.g. evergreen versus deciduous, deep versus shallow roots), and the more contrasting these are, the greater the difference is likely to be. In some cases contrasts may compensate for each other (e.g. evergreen trees with low stomatal conductances versus deciduous trees with high conductances) (Doody *et al*. 2011). In South Africa the most marked contrasts are where short, fairly shallow-rooted, winter-deciduous grasslands are replaced by tall, deep-rooted, evergreen trees (Everson *et al*. 2011). In contrast, invasions by tamarisks in North America have had little impact because they are similar to poplars in their growth form, rooting depth and leaf seasonality (Doody *et al*. 2011; Hultine and Bush 2011).

The 28 invasive taxa in South Africa mapped by Kotzé *et al*. (2010) cover a wide range of growth forms with varying physiology, phenology, rooting depths, LAIs, specific leaf areas and other key traits that affect their potential water-use (Table 1). Even so, they are a subset of the major and emerging invading species in South Africa and elsewhere in the world. However, this review has found that the impacts of the different species do vary in ways that are consistent with their key traits (Fig. 3, Calder 1991, 2005; Le Maitre 2004) so this information can be used for extrapolating the results for known species to unknown species in the interim and to prioritize measurements on other species.

### Stand density and age

The hydrological impacts vary depending on the size (age) (Fig. 3) and density of the invasions with the effects of increasing size and density (canopy cover) being seen in the typical sigmoidal change in streamflow associated with increasing tree or stand age (Bosch and von Gadow 1990; Dye 1996*a*; Le Maitre and Versfeld 1997; Scott *et al*. 2000*a*; Zhao *et al*. 2012). Initially a plantation comprises small saplings, with a low (<1 %) canopy cover, and streamflow reductions are not detectable but, as the trees grow, the reductions become evident with the maximum reductions being reached and sustained following canopy closure, which is well before biomass peaks (Le Maitre and Versfeld 1997). The same structural changes occur as invasions progress (Moody and Mack 1988), so these relationships need to be investigated more fully to allow the effects of size, density and canopy cover to be explicitly included in estimates of the impacts. The same changes occur when native woody plants encroach (i.e. density and canopy cover increases) and insights can be gained from research into the hydrological impacts and controlling factors (e.g. Huxman *et al*. 2005; Wilcox *et al*. 2008).

### Water availability

Invasions occur in two different situations: (i) upland or dryland areas where the available moisture is limited to the rainfall which is retained in the rooting profile and (ii) riparian zones, floodplains or other areas where groundwater is available within the rooting depth. In this second situation, the potential and actual water-use generally is not primarily limited by water availability but by the climatic conditions and the growth form, root depth, phenology and physiology of the plants. More work is needed on riparian invasions both to quantify the impacts of invaders that have not been studied yet and to obtain data on the water-use of the native communities they replace (Salemi *et al*. 2012), or that replace them following clearing.

### Range of climates and invaded vegetation types

Invasions occur across a very wide range of natural vegetation types and climatic conditions so robust guiding principles or rules need to be established for scaling-up from existing measurements to areas where there are no data, especially in semi-arid and arid areas. The ideas discussed by Porporato *et al*. (2004) on seasonality and temporal patterns in rainfall events could be useful in this context. The same considerations apply to situations where more water is available than the soil moisture derived from local rainfall infiltration and percolation. For example, an ephemeral river will have less groundwater available for invaders to use in the long-term than a perennial river system. Remote sensing-based estimates of evaporation provide a tool for addressing many of these issues and ensuring that data from site-specific studies can be scaled up to landscapes and catchments.

## Conclusions

Invasive species do not differ fundamentally from native plant species in their growth forms or physiology. Nevertheless, there are a number of factors that contribute to their greater water-use compared with similar native species, including:

- Plant traits, notably their size, root depths, leaf area or leaf-area index, specific leaf area and transpiration rates. The greatest impacts are found where the invaders are evergreen trees, and the dominant native species are seasonally dormant grasses, but there may be little or no impact where they have similar growth forms and canopy structure (e.g. invasive, deciduous *Tamarix* versus native *Populus* or *Salix* on rivers in the USA). Interestingly, invasive, deciduous *Salix* in Australia can match and, in some settings exceed, the annual water-use of evergreen, native *Eucalyptus* species which are known to have high water-use rates.
- The ability of some invaders to form dense stands compared with co-occurring native species also contributes to disproportionately high stand level water-use by the invasions although the water-use rates by individual species of similar transpiring leaf area maybe similar (S. Dzikiti *et al*., unpubl.).
- The role of the deep root systems of many species which allow them to access soil moisture and groundwater in deep soils and weathered material and in floodplain alluvium where there is additional soil moisture and groundwater (Moore and Heilman 2011).
- This review did not explore the implications of the efficiency with which many of the key invading species produce woody tissues (see Cavaleri and Sack 2010; Funk 2013) and become tall compared with similar native woody species, notably the major genera used in plantations (*Pinus*, *Eucalyptus*, *Acacia*) (Gush and Dye 2009; Wise *et al*. 2011). These are aspects that require further study.

As expected, invaders in dryland settings have a lower water-use than those in floodplains and unconsolidated aquifers and, thus, have less impact on surface runoff per unit area or groundwater. A number of issues require further investigation and addressing them should be a key priority for research on the impacts on invading alien plant species on river flows and groundwater resources.

## Sources of Funding

This work was funded by the Working for Water programme of the Natural Resource Management programmes, Department of Environment Affairs, South Africa.

## Contributions by the Authors

D.C.L.M. led the study and the drafting of the manuscript. M.B.G. and S.D. contributed information, participated in the writing and edited the draft manuscript.

## Conflict of Interest Statement

None declared.

## References

[PLV043C1] Andréassian V 2004 Waters and forests: from historical controversy to scientific debate. Journal of Hydrology 291:1–27. 10.1016/j.jhydrol.2003.12.015

[PLV043C2] Asbjornsen H, Goldsmith GR, Alvarado-Barrientos MS, Rebel K, Van Osch FP, Rietkerk M, Chen J, Gotsch S, Tobon C, Geissert DR, Gomez-Tagle A, Vache K, Dawson TE 2011 Ecohydrological advances and applications in plant-water relations research: a review. Journal of Plant Ecology 4:3–22. 10.1093/jpe/rtr005

[PLV043C3] Asner GP, Scurlock JMO, Hicke JA 2003 Global synthesis of leaf area index observations: implications for ecological and remote sensing studies. Global Ecology and Biogeography 12:191–205. 10.1046/j.1466-822X.2003.00026.x

[PLV043C4] Bastiaanssen WGM, Pelgrum H, Wang J, Ma Y, Moreno JF, Roerink GJ, van der Wal T 1998 A remote sensing surface energy balance algorithm for land (SEBAL). Journal of Hydrology 212–213:213–229. 10.1016/S0022-1694(98)00254-6

[PLV043C5] Benyon RG, Theiveyanathan S, Doody TM 2006 Impacts of tree plantations on groundwater in south-eastern Australia. Australian Journal of Botany 54:181 10.1071/BT05046

[PLV043C6] Bosch JM, Hewlett JD 1982 A review of catchment experiments to determine the effect of vegetation changes on water yield and evapotranspiration. Journal of Hydrology 55:3–23. 10.1016/0022-1694(82)90117-2

[PLV043C7] Bosch JM, von Gadow K 1990 Regulating afforestation for water conservation in South Africa. South African Forestry Journal 153:41–54. 10.1080/00382167.1990.9629032

[PLV043C8] Brown AE, Zhang L, McMahon TA, Western AW, Vertessy RA 2005 A review of paired catchment studies for determining changes in water yield resulting from alterations in vegetation. Journal of Hydrology 310:28–61. 10.1016/j.jhydrol.2004.12.010

[PLV043C9] Budyko MI 1974 Climate and life. International Geophysics Series, Vol. 18 New York: Academic Press.

[PLV043C10a] Bulcock HH, Jewitt GPW. 2010. Spatial mapping of leaf area index using hyperspectral remote sensing for hydrological applications with a particular focus on canopy interception. *Hydrology and Earth System Sciences* **14**:383–392.

[PLV043C10] Bulcock HH, Jewitt GPW 2012 Field data collection and analysis of canopy and litter interception in commercial forest plantations in the KwaZulu-Natal Midlands, South Africa. Hydrology and Earth System Sciences 16:3717–3728. 10.5194/hess-16-3717-2012

[PLV043C11] Calder IR 1991 Water use by forests at the plot and catchment scale. Commonwealth Forestry Review 75:19–30.

[PLV043C12] Calder IR 2005 The blue revolution: land use and integrated water resources management. London: Earthscan Publications.

[PLV043C13] Calder IR, Dye P 2001 Hydrological impacts of invasive alien plants. Land Use and Water Resources Research 1:8.2–8.12.

[PLV043C14] Canadell J, Jackson RB, Ehleringer JB, Mooney HA, Sala OE, Schulze ED 1996 Maximum rooting depth of vegetation types at the global scale. Oecologia 108:583–595. 10.1007/BF00329030 28307789

[PLV043C15] Carter JL, White DA 2009 Plasticity in the Huber value contributes to homeostasis in leaf water relations of a mallee Eucalypt with variation to groundwater depth. Tree Physiology 29:1407–1418. 10.1093/treephys/tpp076 19797243

[PLV043C16] Cavaleri MA, Sack L 2010 Comparative water use of native and invasive plants at multiple scales: a global meta-analysis. Ecology 91:2705–2715. 10.1890/09-0582.1 20957964

[PLV043C17] Cleugh HA, Leuning R, Mu Q, Running SW 2007 Regional evaporation estimates from flux tower and MODIS satellite data. Remote Sensing of Environment 106:285–304. 10.1016/j.rse.2006.07.007

[PLV043C18] Cleverly JR, Dahm CN, Thibault JR, McDonnell DE, Coonrod JEA 2006 Riparian ecohydrology: regulation of water flux from the ground to the atmosphere in the Middle Rio Grande, New Mexico. Hydrological Processes 20:3207–3225. 10.1002/hyp.6328

[PLV043C19] Clulow AD, Everson CS, Gush MB 2011 The long-term impact of Acacia mearnsii trees on evaporation, streamflow and groundwater resources. Report No. TT 505/11 Pretoria: Water Research Commission.

[PLV043C20] Cullis JDS, Görgens AHM, Marais C 2007 A strategic study of the impact of invasive alien plants in the high rainfall catchments and riparian zones of South Africa on total surface water yield. Water SA 33:35–42.

[PLV043C21] Dahm CN, Cleverly JR, Coonrod JEA, Thibault JR, McDonnell DE, Gilroy DJ 2002 Evapotranspiration at the land/water interface in a semi-arid drainage basin. Freshwater Biology 47:831–843. 10.1046/j.1365-2427.2002.00917.x

[PLV043C22] Dawson TE, Pate JS 1996 Seasonal water uptake and movement in root systems of Australian phraeatophytic plants of dimorphic root morphology: a stable isotope investigation. Oecologia 107:13–20. 10.1007/BF00582230 28307187

[PLV043C23] Diaz S, Hodgson JG, Thompson K, Cabido M, Cornelissen JHC, Jalili A, Montserrat-Martí G, Grime JP, Zarrinkamar F, Asri Y, Band SR, Basconcelo S, Castro-Díez P, Funes G, Hamzehee B, Khoshnevi M, Pérez-Harguindeguy N, Pérez-Rontomé MC, Shirvany FA, Vendramini F, Yazdani S, Abbas-Azimi R, Bogaard A, Boustani S, Charles M, Dehghan M, de Torres-Espuny L, Falczuk V, Guerrero-Campo J, Hynd A, Jones G, Kowsary E, Kazemi-Saeed F, Maestro-Martínez M, Romo-Díez A, Shaw S, Siavash B, Villar-Salvador P, Zak MR 2004 The plant traits that drive ecosystems: evidence from three continents. Journal of Vegetation Science 15:295–304. 10.1111/j.1654-1103.2004.tb02266.x

[PLV043C24] Dickie IA, Bennett BM, Burrows LE, Nuñez MA, Peltzer DA, Porté A, Richardson DM, Rejmánek M, Rundel PW, van Wilgen BW 2014 Conflicting values: ecosystem services and invasive tree management. Biological Invasions 16:705–719. 10.1007/s10530-013-0609-6

[PLV043C25] Dillon P, Benyon R, Cook P, Hatton T, Marvanek S, Gillooly J 2001 Review of research on plantation forest water requirements in relation to groundwater resources in the southeast of South Australia. Report No. 99 Urrbrae, South Australia: Centre for Groundwater Studies.

[PLV043C26] Donohue RJ, Roderick ML, McVicar TR 2010 Can dynamic vegetation information improve the accuracy of Budyko's hydrological model? Journal of Hydrology 390:23–34. 10.1016/j.jhydrol.2010.06.025

[PLV043C27] Donohue RJ, Roderick ML, McVicar TR 2012 Roots, storms and soil pores: incorporating key ecohydrological processes into Budyko's hydrological model. Journal of Hydrology 436–437:35–50. 10.1016/j.jhydrol.2012.02.033

[PLV043C28] Doody T, Benyon R 2011 Quantifying water savings from willow removal in Australian streams. Journal of Environmental Management 92:926–935. 10.1016/j.jenvman.2010.10.061 21106290

[PLV043C29] Doody TM, Nagler PL, Glenn EP, Moore GW, Morino K, Hultine KR, Benyon RG 2011 Potential for water salvage by removal of non-native woody vegetation from dryland river systems. Hydrological Processes 25:4117–4131. 10.1002/hyp.8395

[PLV043C30] Doody TM, Benyon RG, Theiveyanathan S, Koul V, Stewart L 2014 Development of pan coefficients for estimating evapotranspiration from riparian woody vegetation. Hydrological Processes 28:2129–2149. 10.1002/hyp.9753

[PLV043C31] Dovey S 2005 Above-ground allometry, biomass and nutrient content of Acacia mearnsii across four ages and three sites in the KwaZulu-Natal Midlands. MSc Thesis, University of KwaZulu-Natal, Pietermaritzburg.

[PLV043C32] Drenovsky RE, Grewell BJ, D'Antonio CM, Funk JL, James JJ, Molinari N, Parker IM, Richards CL 2012 A functional trait perspective on plant invasion. Annals of Botany 110:141–153. 10.1093/aob/mcs100 22589328 PMC3380596

[PLV043C33] Dye PJ 1996a Climate, forest and streamflow relationships in South African afforested catchments. Commonwealth Forestry Review 75:31–38.

[PLV043C34] Dye PJ 1996b Response of *Eucalyptus grandis* trees to soil water deficits. Tree Physiology 16:233–238. 10.1093/treephys/16.1-2.233 14871767

[PLV043C35] Dye P, Jarmain C 2004 Water use by black wattle (*Acacia mearnsii*): implications for the link between removal of invading trees and catchment streamflow response. South African Journal of Science 100:40–44.

[PLV043C36] Dye PJ, Poulter AG 1993 A field demonstration of the effect on streamflow of clearing invasive pine and wattle trees from a riparian zone. South African Forestry Journal 173:27–30.

[PLV043C37a] Dye PJ, Poulter AG, Hudson K, Soko S. 1996. *A comparison of the water use of common riparian forests and grasslands*. CSIR Report FOR-DEA 925. CSIR, Pretoria: Division of Forest Science and Technology.

[PLV043C37] Dye PJ, Jarmain C, Le Maitre DC, Everson C, Gush MB, Clulow A 2008 Modeling vegetation water use for general application in different categories of vegetation. Report 1319/1/08 Pretoria: Water Research Commission.

[PLV043C38] Dzikiti S, Schachtschneider K, Naiken V, Gush M, Le Maitre D 2013a Comparison of water-use by alien invasive pine trees growing in riparian and non-riparian zones in the Western Cape Province, South Africa. Forest Ecology and Management 293:92–102. 10.1016/j.foreco.2013.01.003

[PLV043C39] Dzikiti S, Schachtschneider K, Naiken V, Gush M, Moses G, Le Maitre DC 2013b Water relations and the effects of clearing invasive Prosopis trees on groundwater in an arid environment in the Northern Cape, South Africa. Journal of Arid Environments 90:103–113. 10.1016/j.jaridenv.2012.10.015

[PLV043C40] Eagleson PS 1978 Climate, soil, and vegetation: 1. Introduction to water balance dynamics. Water Resources Research 14:705–712. 10.1029/WR014i005p00705

[PLV043C41] Ehrenfeld JG 2010 Ecosystem consequences of biological invasions. Annual Review of Ecology, Evolution, and Systematics 41:59–80. 10.1146/annurev-ecolsys-102209-144650

[PLV043C42] Engel V, Jobbágy EG, Stieglitz M, Williams M, Jackson RB 2005 Hydrological consequences of Eucalyptus afforestation in the Argentine Pampas. Water Resources Research 41:W10409 10.1029/2004WR003761

[PLV043C43] Enloe SF, DiTomaso JM, Orloff SB, Drake DJ 2004 Soil water dynamics differ among rangeland plant communities dominated by yellow starthistle (*Centaurea solstitialis*), annual grasses, or perennial grasses. Weed Science 52:929–935. 10.1614/WS-03-156R

[PLV043C44] Enquist BJ, West GB, Charnov EL, Brown JH 1999 Allometric scaling of production and life-history variation in vascular plants. Nature 401:907–911. 10.1038/44819

[PLV043C45] Everson C 1999 Evaporation from the Orange River: quantifying open water resources. Report No. 683/1/99 Pretoria, South Africa: Water Research Commission.

[PLV043C46] Everson C, Burger C, Olbrich BW, Gush MB 2001 Verification of estimates of water use from riparian vegetation on the Sabie River in the Kruger National Park. Report no. 877/1/01 Pretoria, South Africa: Water Research Commission.

[PLV043C47] Everson CS, Gush MB, Moodley M, Jarmain C, Govender M, Dye P 2007 Effective management of the riparian zone vegetation to significantly reduce the cost of catchment management and enable greater productivity of land resources. Report No. 1284/1/07 Pretoria: Water Research Commission.

[PLV043C48] Everson C, Clulow A, Mengistu M 2009 Feasibility study on the determination of riparian vegetation evaporation on on-perennial systems. Report No. TT 424/09 Pretoria, South Africa: Water Research Commission.

[PLV043C49] Everson CS, Dye PJ, Gush MB, Everson TM 2011 Water use of grasslands, agroforestry systems and indigenous forests. Water SA 37:781–788. 10.4314/wsa.v37i5.15

[PLV043C50] Farley KA, Jobbágy EG, Jackson RB 2005 Effects of afforestation on water yield: a global synthesis with implications for policy. Global Change Biology 11:1565–1576. 10.1111/j.1365-2486.2005.01011.x

[PLV043C51] Fink KA, Wilson SD 2011 *Bromus inermis* invasion of a native grassland: diversity and resource reduction. Botany 89:157–164. 10.1139/B11-004

[PLV043C52] Forsyth GG, Le Maitre DC, O'Farrell PJ, van Wilgen BW 2012 The prioritisation of invasive alien plant control projects using a multi-criteria decision model informed by stakeholder input and spatial data. Journal of Environmental Management 103:51–57. 10.1016/j.jenvman.2012.01.034 22459070

[PLV043C53] Fourie F, Mbatha K, Verster H, Van Dyk G 2007 The effect of vegetation (Prosopis sp.) on groundwater levels in Rugseer River, Kenhardt, South Africa. *Groundwater and Ecosystems, XXXV IAH Congress* Poster paper presented at Groundwater and Ecosystems, XXXV IAH Congress, 17–21 September 2007, Lisbon, Portugal, 8.

[PLV043C54] Fritzsche F, Abate A, Fetene M, Beck E, Weise S, Guggenberger G 2006 Soil-plant hydrology of indigenous and exotic trees in an Ethiopian montane forest. Tree Physiology 26:1043–1054. 10.1093/treephys/26.8.1043 16651254

[PLV043C55] Funk JL 2013 The physiology of invasive plants in low-resource environments. Conservation Physiology 1:10.1093/conphys/cot026. PMC4806624 27293610

[PLV043C56] Funk JL, Matzek V, Bernhardt M, Johnson D 2014 Broadening the case for invasive species management to include impacts on ecosystem services. BioScience 64:58–63. 10.1093/biosci/bit004

[PLV043C57a] Gazal RM, Scott RL, Goodrich DC, Williams DG. 2006. Controls on transpiration in a semiarid riparian cottonwood forest. *Agricultural and Forest Meteorology* **137**:56–67.

[PLV043C57] Gerlach JD 2004 The impacts of serial land-use changes and biological invasions on soil water resources in California, USA. Journal of Arid Environments 57:365–379. 10.1016/S0140-1963(03)00102-2

[PLV043C58] Glenn EP, Nagler PL 2005 Comparative ecophysiology of Tamarix ramosissima and native trees in western U.S. riparian zones. Journal of Arid Environments 61:419–446. 10.1016/j.jaridenv.2004.09.025

[PLV043C59] Glenn EP, Neale CMU, Hunsaker DJ, Nagler PL 2011 Vegetation index-based crop coefficients to estimate evapotranspiration by remote sensing in agricultural and natural ecosystems. Hydrological Processes 25:4050–4062. 10.1002/hyp.8392

[PLV043C60] Görgens AHM, van Wilgen BW 2004 Invasive alien plants and water resources in South Africa: current understanding, predictive ability and research challenges. South African Journal of Science 100:27–33.

[PLV043C61] Gower ST, Kucharik CJ, Norman JM 1999 Direct and indirect estimation of leaf area index, fAPAR, and net primary production of terrestrial ecosystems. Remote Sensing of Environment 70:29–51. 10.1016/S0034-4257(99)00056-5

[PLV043C62] Grotkopp E, Rejmánek M 2007 High seedling relative growth rate and specific leaf area are traits of invasive species: phylogenetically independent contrasts of woody angiosperms. American Journal of Botany 94:526–532. 10.3732/ajb.94.4.526 21636422

[PLV043C63] Grotkopp E, Rejmánek M, Rost TL 2002 Toward a causal explanation of plant invasiveness: seedling growth and life-history strategies of 29 pine (*Pinus*) species. The American Naturalist 159:396–419. 10.1086/338995 18707424

[PLV043C64] Gush MB 2011 Water-use, growth and water-use efficiency of indigenous tree species in a range of forest and woodland systems in South Africa. Unpublished PhD Thesis, University of Cape Town.

[PLV043C65] Gush MB, Dye PJ 2009 Water-use efficiency within a selection of indigenous and exotic tree species in South Africa as determined using sap flow and biomass measurements. Acta Horticulturae 846:323–330.

[PLV043C66] Haigh H 1966 Root development in the sandy soils of Zululand. Forestry in South Africa 7:31–36.

[PLV043C67] Hatton T, Reece P, Taylor P, McEwan K 1998 Does leaf water efficiency vary among eucalypts in water-limited environments? Tree Physiology 18:529–536. 10.1093/treephys/18.8-9.529 12651339

[PLV043C68a] Henderson L. 2001. *Alien weeds and invasive plants*. Plant Protection Institute Handbook No. 12. Pretoria: Agricultural Research Council.

[PLV043C68] Hulme PE, Pyšek P, Jarošík V, Pergl J, Schaffner U, Vilà M 2013 Bias and error in understanding plant invasion impacts. Trends in Ecology & Evolution 28:212–218. 10.1016/j.tree.2012.10.010 23153723

[PLV043C69] Hultine KR, Bush SE 2011 Ecohydrological consequences of non-native riparian vegetation in the southwestern United States: a review from an ecophysiological perspective. Water Resources Research 47:W07542 10.1029/2010WR010317

[PLV043C70] Huxman TE, Wilcox BP, Breshears DD, Scott RL, Snyder KA, Small EE, Hultine K, Pockman WT, Jackson RB 2005 Ecohydrological implications of woody plant encroachment. Ecology 86:308–319. 10.1890/03-0583

[PLV043C71] Jackson RB, Canadell J, Ehleringer JR, Mooney HA, Sala OE, Schulze ED 1996 A global analysis of root distributions for terrestrial biomes. Oecologia 108:389–411. 10.1007/BF00333714 28307854

[PLV043C72] Jarmain C, Everson C 2002 Comparative evaporation measurements above commercial forestry and sugar cane canopies in the KwaZulu-Natal Midlands. Report to Department of Water Affairs and Forestry, Division of Water, Environment and Forestry Technology Pietermaritzburg: CSIR.

[PLV043C73] Jarmain C, Meijninger W 2012 Assessing the impact of invasive alien plants on South African water resources using remote sensing techniques. Proceedings of a symposium held at Jackson Hole*,* Wyoming, USA*,* September 2010 *Remote Sensing and Hydrology*. IAHS Publication No 352, IAHS, Wallingford, UK, 388–393.

[PLV043C75] Jobbágy EG, Jackson RB 2004 Groundwater use and salinization with grassland afforestation. Global Change Biology 10:1299–1312. 10.1111/j.1365-2486.2004.00806.x

[PLV043C76] Kagawa A, Sack L, Duarte K, James S 2009 Hawaiian native forest conserves water relative to timber plantation: species and stand traits influence water use. Ecological Applications 19:1429–1443. 10.1890/08-1704.1 19769092

[PLV043C77] Kiniry JR 1998 Biomass accumulation and radiation use efficiency of honey mesquite and eastern red cedar. Biomass and Bioenergy 15:467–473. 10.1016/S0961-9534(98)00057-9

[PLV043C78] Komatsu H, Cho J, Matsumoto K, Otsuki K 2012 Simple modeling of the global variation in annual forest evapotranspiration. Journal of Hydrology 420–421:380–390. 10.1016/j.jhydrol.2011.12.030

[PLV043C79] Kotzé I, Beukes H, van den Berg E, Newby T 2010 National Invasive Alien Plant Survey. Report No. GW/A/2010/21 Pretoria: Agricultural Research Council—Institute for Soil, Climate and Water.

[PLV043C80] Kruger FJ 1977 Invasive woody plants in Cape fynbos with special reference to the biology and control of *Pinus pinaster*. Proceedings of the Second National Weeds Conference of South Africa Weed Society, 54–74.

[PLV043C81] Kruger FJ, Bennett BM 2013 Wood and water: an historical assessment of South Africa's past and present forestry policies as they relate to water conservation. Transactions of the Royal Society of South Africa 68:163–174. 10.1080/0035919X.2013.833144

[PLV043C82] Lacey JR, Marlow CB, Lane JR, Marlow B 1989 Influence of spotted knapweed (*Centaurea maculosa*) on surface runoff and sediment yield. Weed Technology 3:627–631.

[PLV043C83] Landsberg JJ, Waring RH 1997 A generalised model of forest productivity using simplified concepts of radiation-use efficiency, carbon balance and partitioning. Forest Ecology and Management 95:209–228. 10.1016/S0378-1127(97)00026-1

[PLV043C84] Larcher W 1975 Physiological plant ecology. Berlin: Springer.

[PLV043C85] Lavorel S, Garnier E 2002 Predicting changes in community composition and ecosystem functioning from plant traits: revisiting the Holy Grail. Functional Ecology 16:545–556. 10.1046/j.1365-2435.2002.00664.x

[PLV043C86] Lavorel S, McIntyre S, Landsberg J, Forbes TDA 1997 Plant functional classifications: from general groups to specific groups based on response to disturbance. Trends in Ecology and Evolution 12:474–478. 10.1016/S0169-5347(97)01219-6 21238163

[PLV043C87] Leishman MR, Haslehurst T, Ares A, Baruch Z 2007 Leaf trait relationships of native and invasive plants: community- and global-scale comparisons. New Phytologist 176:635–643. 10.1111/j.1469-8137.2007.02189.x 17822409

[PLV043C88] Le Maitre DC 1999 Prosopis and groundwater: a literature review and bibliography. Report No. ENV-S-C 99077, Environmentek, CSIR. Unpublished Report, Working for Water Programme, Department of Water Affairs and Forestry.

[PLV043C89] Le Maitre DC 2004 Predicting invasive species impacts on hydrological processes: the consequences of plant physiology for landscape processes. Weed Technology 18:1408–1410. 10.1890/0012-9615(2002)072[0311:TGBOR]2.0.CO;2

[PLV043C90] Le Maitre DC, Versfeld DB 1997 Forest evaporation models: relationships between stand growth and evaporation. Journal of Hydrology 193:240–257. 10.1016/S0022-1694(96)03144-7

[PLV043C91] Le Maitre DC, van Wilgen BW, Chapman RA, McKelly DH 1996 Invasive plants and water resources in the Western Cape Province, South Africa: modelling the consequences of a lack of management. Journal of Applied Ecology 33:161–172. 10.2307/2405025

[PLV043C92] Le Maitre DC, Versfeld DB, Chapman RA 2000 The impact of invading alien plants on surface water resources in South Africa: a preliminary assessment. Water SA 26:397–408.

[PLV043C93] Levine JM, Vila M, D'Antonio CM, Dukes JS, Grigulis K, Lavorel S 2003 Mechanisms underlying the impacts of exotic plant invasions. Proceedings of the Royal Society of London Series B: Biological Sciences 270:775–781. 10.1098/rspb.2003.2327 12737654 PMC1691311

[PLV043C94] McDowell N, Barnard H, Bond B, Hinckley T, Hubbard R, Ishii H, Köstner B, Magnani F, Marshall J, Meinzer F, Phillips N, Ryan M, Whitehead D 2002 The relationship between tree height and leaf area: sapwood area ratio. Oecologia 132:12–20. 10.1007/s00442-002-0904-x 28547290

[PLV043C95] McElrone AJ, Pockman WT, Martínez-Vilalta J, Jackson RB 2004 Variation in xylem structure and function in stems and roots of trees to 20 m depth. New Phytologist 163:507–517. 10.1111/j.1469-8137.2004.01127.x 33873731

[PLV043C96] Meijninger W, Jarmain C 2009 Development of remote sensing tools for monitoring the hydrological benefits of the Working for Water Program. Cape Town: Report prepared for the Working for Water Programme, Working for Water, Cape Town.

[PLV043C97] Meijninger WML, Jarmain C 2014 Satellite-based annual evaporation estimates of invasive alien plant species and native vegetation in South Africa. Water SA 40:95–107. 10.4314/wsa.v40i1.12

[PLV043C98] Mendham DS, White DA, Battaglia M, McGrath JF, Short TM, Ogden GN, Kinal J 2011 Soil water depletion and replenishment during first- and early second-rotation Eucalyptus globulus plantations with deep soil profiles. Agricultural and Forest Meteorology 151:1568–1579. 10.1016/j.agrformet.2011.06.014

[PLV043C99] Moles AT, Ackerly DD, Webb CO, Tweddle JC, Dickie JB, Pitman AJ, Westoby M 2005 Factors that shape seed mass evolution. Proceedings of the National Academy of Sciences of the USA 102:10540–10544. 10.1073/pnas.0501473102 16030149 PMC1180762

[PLV043C100] Moles AT, Flores-Moreno H, Bonser SP, Warton DI, Helm A, Warman L, Eldridge DJ, Jurado E, Hemmings FA, Reich PB, Cavender-Bares J, Seabloom EW, Mayfield MM, Sheil D, Djietror JC, Peri PL, Enrico L, Cabido MR, Setterfield SA, Lehmann CER, Thomson FJ 2012 Invasions: the trail behind, the path ahead, and a test of a disturbing idea. Journal of Ecology 100:116–127. 10.1111/j.1365-2745.2011.01915.x

[PLV043C101] Moody ME, Mack RN 1988 Controlling the spread of plant invasions: the importance of nascent foci. Journal of Applied Ecology 25:1009–1021. 10.2307/2403762

[PLV043C102] Moore GW, Heilman JL 2011 Proposed principles governing how vegetation changes affect transpiration. Ecohydrology 4:351–358. 10.1002/eco.232

[PLV043C103] Moore GW, Owens MK 2012 Transpirational water loss in invaded and restored semiarid riparian forests. Restoration Ecology 20:346–351. 10.1111/j.1526-100X.2011.00774.x

[PLV043C104] Morris TL, Esler KJ, Barger NN, Jacobs SM, Cramer MD 2011 Ecophysiological traits associated with the competitive ability of invasive Australian acacias. Diversity and Distributions 17:898–910. 10.1111/j.1472-4642.2011.00802.x

[PLV043C105] Mu Q, Zhao M, Running SW 2011 Improvements to a MODIS global terrestrial evapotranspiration algorithm. Remote Sensing of Environment 115:1781–1800. 10.1016/j.rse.2011.02.019

[PLV043C106] Mucina L, Rutherford MC (eds.) 2006 The vegetation of South Africa, Lesotho and Swaziland. Strelitzia 19. Pretoria: South African National Biodiversity Institute.

[PLV043C107] Nagler PL, Cleverly J, Glenn E, Lampkin D, Huete A, Wan Z 2005a Predicting riparian evapotranspiration from MODIS vegetation indices and meteorological data. Remote Sensing of Environment 94:17–30. 10.1016/j.rse.2004.08.009

[PLV043C108] Nagler PL, Scott RL, Westenburg C, Cleverly JR, Glenn EP, Huete AR 2005b Evapotranspiration on western U.S. rivers estimated using the Enhanced Vegetation Index from MODIS and data from eddy covariance and Bowen ratio flux towers. Remote Sensing of Environment 97:337–351. 10.1016/j.rse.2005.05.011

[PLV043C109] Nagler PL, Morino K, Didan K, Erker J, Osterberg J, Hultine KR, Glenn EP 2009 Wide-area estimates of saltcedar (Tamarix spp.) evapotranspiration on the lower Colorado River measured by heat balance and remote sensing methods. Ecohydrology 2:18–33. 10.1002/eco.35

[PLV043C110] Nagler PL, Shafroth PB, Labaugh JW, Snyder KA, Scott RL, Merritt DM, Osterberg J 2010 The potential for water savings through the control of Saltcedar and Russian Olive. In: Shafroth PB, Brown CA, Merritt DM, eds. Saltcedar and Russian Olive control demonstration act science assessment. Scientific Investigations Report 2009-5247 Reston, VA: U.S. Geological Survey, 35–47.

[PLV043C111] Niklas KJ, Midgley JJ, Enquist BJ 2003 A general model for mass—growth—density relations across tree-dominated communities. Evolutionary Ecology Research 5:459–468.

[PLV043C112] O'Grady AP, Carter JL, Bruce J 2011 Can we predict groundwater discharge from terrestrial ecosystems using existing eco-hydrological concepts? Hydrology and Earth System Sciences 15:3731–3739. 10.5194/hess-15-3731-2011

[PLV043C113] Oliveira RS, Dawson TE, Burgess SSO, Nepstad DC 2005 Hydraulic redistribution in three Amazonian trees. Oecologia 145:354–363. 10.1007/s00442-005-0108-2 16091971

[PLV043C114] Ordonez A, Wright IJ, Olff H 2010 Functional differences between native and alien species: a global-scale comparison. Functional Ecology 24:1353–1361. 10.1111/j.1365-2435.2010.01739.x

[PLV043C115] Pate JS, Jeschke WD, Aylward MJ 1995 Hydraulic architecture and xylem structure of the dimorphic root systems of South-West Australian species of Proteaceae. Journal of Experimental Botany 46:907–915. 10.1093/jxb/46.8.907

[PLV043C116] Poorter H, Niklas KJ, Reich PB, Oleksyn J, Poot P, Mommer L 2012 Tansley review Biomass allocation to leaves, stems and roots: meta-analyses of interspecific variation and environmental control. New Phytologist 193:30–50. 22085245 10.1111/j.1469-8137.2011.03952.x

[PLV043C117] Porporato A, Rodriguez-Iturbe I 2013 From random variability to ordered structures: a search for general synthesis in ecohydrology. Ecohydrology 6:333–342. 10.1002/eco.1400

[PLV043C118] Porporato A, Daly E, Rodriguez-Iturbe I 2004 Soil water balance and ecosystem response to climate change. The American Naturalist 164:625–632. 10.1086/424970 15540152

[PLV043C119] Potter NJ, Zhang L, Milly PCD, McMahon TA, Jakeman AJ 2005 Effects of rainfall seasonality and soil moisture capacity on mean annual water balance for Australian catchments. Water Resources Research 41:W06007 10.1029/2004WR003697

[PLV043C120] Prinsloo FW, Scott DF 1999 Streamflow responses to the clearing of alien invasive trees from riparian zones at three sites in the Western Cape Province. Southern African Forestry Journal 185:1–7. 10.1080/10295925.1999.9631220

[PLV043C121] Pyšek P, Richardson DM 2010 Invasive species, environmental change and management, and health. Annual Review of Environment and Resources 35:25–55. 10.1146/annurev-environ-033009-095548

[PLV043C122] Pyšek P, Jarošík V, Hulme PE, Pergl J, Hejda M, Schaffner U, Vilà M 2012 A global assessment of invasive plant impacts on resident species, communities and ecosystems: the interaction of impact measures, invading species’ traits and environment. Global Change Biology 18:1725–1737. 10.1111/j.1365-2486.2011.02636.x

[PLV043C123] Read C, Wright IJ, Westoby M 2006 Scaling-up from leaf to canopy-aggregate properties in sclerophyll shrub species. Austral Ecology 31:310–316. 10.1111/j.1442-9993.2006.01559.x

[PLV043C124] Reich PB, Walters MB, Ellsworth DS 1997 From tropics to tundra: global convergence in plant functioning. Proceedings of the National Academy of Sciences of the USA 94:13730–13734. 10.1073/pnas.94.25.13730 9391094 PMC28374

[PLV043C125] Rejmánek M, Richardson DM 2013 Trees and shrubs as invasive alien species—2013 update of the global database. Diversity and Distributions 19:1093–1094. 10.1111/ddi.12075

[PLV043C126] Richardson DM, Cambray JA, Chapman RA, Dean WRJ, Griffiths CL, Le Maitre DC, Newton DJ, Winstanley T 2003 Vectors and pathways of biological invasions in South Africa—past, present, and future. Invasive species: vectors and management strategies. Island Press, 292–349.

[PLV043C127] Rodriguez-Iturbe I, Porporato A, Ridolfi L, Isham V, Coxi DR 1999 Probabilistic modelling of water balance at a point: the role of climate, soil and vegetation. Proceedings of the Royal Society of London. Series A: Mathematical, Physical and Engineering Sciences 455:3789–3805. 10.1098/rspa.1999.0477

[PLV043C128] Rowntree KM, Beyers GJ 1999 An experimental study of the effect of Acacia mearnsii on streamflow in the Sand River, Zwartkops River catchment, Eastern Cape. Report KV 123/99 Pretoria: Water Research Commission.

[PLV043C129] Running SW, Coughlan JC 1988 A general model of forest ecosystem processes for regional applications I. Hydrologic balance, canopy gas exchange and primary production processes. Ecological Modelling 42:125–154. 10.1016/0304-3800(88)90112-3

[PLV043C130] Sala A, Smith SD, Devit DA 1996 Water use by Tamarix ramosissima and associated phreatophytes in a Mojave Desert floodplain. Ecological Applications 6:888–898. 10.2307/2269492

[PLV043C131] Salemi LF, Groppo JD, Trevisan R, Marcos de Moraes J, de Paula Lima W, Martinelli LA 2012 Riparian vegetation and water yield: a synthesis. Journal of Hydrology 454–455:195–202. 10.1016/j.jhydrol.2012.05.061

[PLV043C132] Schenk HJ, Jackson RB 2002a The global biogeography of roots. Ecological Monographs 72:311–328. 10.1890/0012-9615(2002)072[0311:TGBOR]2.0.CO;2

[PLV043C133] Schenk HJ, Jackson RB 2002b Rooting depths, lateral root spreads and below-ground/above-ground allometries of plants in water-limited ecosystems. Journal of Ecology 90:480–494. 10.1046/j.1365-2745.2002.00682.x

[PLV043C134a] Schulze RE. 1979. *Hydrology and water resources of the Drakensberg*. Pietermaritzburg, South Africa: Natal Town and Regional Planning Commission.

[PLV043C134] Scott DF 1999 Managing riparian zone vegetation to sustain streamflow: results of paired catchment experiments in South Africa. Canadian Journal of Forest Research 29:1149–1157. 10.1139/x99-042

[PLV043C135] Scott DF, Lesch W 1997 Streamflow responses to afforestation with *Eucalyptus grandis* and *Pinus patula* and to felling in the Mokobulaan experimental catchments, South Africa. Journal of Hydrology 199:360–377. 10.1016/S0022-1694(96)03336-7

[PLV043C136] Scott DF, Smith RE 1997 Preliminary empirical models to predict reductions in total and low flows resulting from afforestation. Water SA 23:135–140.

[PLV043C137] Scott DF, Prinsloo FW, Moses G, Mehlomakulu M, Simmers ADA 2000a A re-analysis of the South African catchment afforestation experimental data. Report No. 810/1/00 Pretoria: Water Research Commission.

[PLV043C139] Scott DF, Bruijnzeel LA, Vertessy R, Calder IR 2004 Forest hydrology: impacts of forest plantations on streamflow. In: Burley J, Evans J, Youngquist JA, eds. The encyclopedia of forest sciences. Oxford: Elsevier, 367–377.

[PLV043C138] Scott RL, Shuttleworth WJ, Goodrich DC, Maddock T III 2000b The water use of two dominant vegetation communities in a semiarid riparian ecosystem. Agricultural and Forest Meteorology 105:241–256. 10.1016/S0168-1923(00)00181-7

[PLV043C140] Scott RL, Edwards EA, Shuttleworth WJ, Huxman TE, Watts C, Goodrich DC 2004 Interannual and seasonal variation in fluxes of water and carbon dioxide from a riparian woodland ecosystem. Agricultural and Forest Meteorology 122:65–84. 10.1016/j.agrformet.2003.09.001

[PLV043C141] Scott RL, Huxman TE, Williams DG, Goodrich DC 2006 Ecohydrological impacts of woody-plant encroachment: seasonal patterns of water and carbon dioxide exchange within a semiarid riparian environment. Global Change Biology 12:311–324. 10.1111/j.1365-2486.2005.01093.x

[PLV043C142] Scott RL, Cable WL, Huxman TE, Nagler PL, Hernandez M, Goodrich DC 2008 Multiyear riparian evapotranspiration and groundwater use for a semiarid watershed. Journal of Arid Environments 72:1232–1246. 10.1016/j.jaridenv.2008.01.001

[PLV043C143] Sharifi MR, Nilsen ET, Rundel PW 1982 Biomass and net primary production of Prosopis glandulosa (Fabaceae) in the Sonoran Desert of California. American Journal of Botany 69:760–767. 10.2307/2442966

[PLV043C144] Slaats JJP, Van Der Heiden WM, Stockmann CM, Wessel M, Janssen BH 1996 Growth of the *Chromolaena odorata* fallow vegetation in semi-permanent food crop production systems in south-west Cote d'Ivoire. Netherlands Journal of Agricultural Science 44:179–182.

[PLV043C145] Stromberg JC 2013 Root patterns and hydrogeomorphic niches of riparian plants in the American Southwest. Journal of Arid Environments 94:1–9. 10.1016/j.jaridenv.2013.02.004

[PLV043C146] Tecco PA, Díaz S, Cabido M, Urcelay C 2010 Functional traits of alien plants across contrasting climatic and land-use regimes: do aliens join the locals or try harder than them? Journal of Ecology 98:17–27. 10.1111/j.1365-2745.2009.01592.x

[PLV043C147] Tecco PA, Urcelay C, Dìaz S, Cabido M, Pérez-Harguindeguy N 2013 Contrasting functional trait syndromes underlay woody alien success in the same ecosystem. Austral Ecology 38:443–451. 10.1111/j.1442-9993.2012.02428.x

[PLV043C148] Thornthwaite CW 1948 An approach toward a rational classification of climate. Geographical Review 38:55–94. 10.2307/210739

[PLV043C149] Tyree MT, Sperry JS 1988 Do woody plants operate near the point of catastrophic xylem dysfunction caused by dynamic water stress?: answers from a model. Plant Physiology 88:574–580. 10.1104/pp.88.3.574 16666351 PMC1055627

[PLV043C150] USDA NRCS. 2013 The PLANTS Database. Greensboro: National Plant Data Team http://plants.usda.gov (November 2013).

[PLV043C151] Van Bodegom PM, Douma JC, Witte JPM, Ordoñez JC, Bartholomeus RP, Aerts R 2012 Going beyond limitations of plant functional types when predicting global ecosystem-atmosphere fluxes: exploring the merits of traits-based approaches. Global Ecology and Biogeography 21:625–636. 10.1111/j.1466-8238.2011.00717.x

[PLV043C152] Van den Berg E 2010 Detection, quantification and monitoring Prosopis spp. in the Northern Cape Province of South Africa using remote sensing and GIS. MSc Thesis, University of the North-West, Potchefstroom.

[PLV043C153] van Laar A 1983 A case study in Populus canescens to estimate the leaf area index. South African Forestry Journal 125:80–84. 10.1080/00382167.1983.9628880

[PLV043C154] van Laar A 1984 The estimation of the leaf area index for a mature *Pinus radiata* stand. South African Forestry Journal 128:8–11. 10.1080/00382167.1984.9628918

[PLV043C155] van Wilgen BW, Richardson DM 2014 Challenges and trade-offs in the management of invasive alien trees. Biological Invasions 16:721–734. 10.1007/s10530-013-0615-8

[PLV043C156] van Wilgen BW, Le Maitre DC, Cowling RM 1998 Ecosystem services, efficiency, sustainability and equity: South Africa's Working for Water Programme. Trends in Ecology & Evolution 13:378 10.1016/S0169-5347(98)01434-7 21238351

[PLV043C157] van Wilgen BW, Reyers B, Le Maitre DC, Richardson DM, Schonegevel L 2008 A biome-scale assessment of the impact of invasive alien plants on ecosystem services in South Africa. Journal of Environmental Management 89:336–349. 10.1016/j.jenvman.2007.06.015 17765388

[PLV043C158] Velpuri NM, Senay GB, Singh RK, Bohms S, Verdin JP 2013 A comprehensive evaluation of two MODIS evapotranspiration products over the conterminous United States: using point and gridded FLUXNET and water balance ET. Remote Sensing of Environment 139:35–49. 10.1016/j.rse.2013.07.013

[PLV043C159] Vilà M, Espinar JL, Hejda M, Hulme PE, Jarošík V, Maron JL, Pergl J, Schaffner U, Sun Y, Pyšek P 2011 Ecological impacts of invasive alien plants: a meta-analysis of their effects on species, communities and ecosystems. Ecology Letters 14:702–708. 10.1111/j.1461-0248.2011.01628.x 21592274

[PLV043C160] Waterloo MJ, Bruijnzeel LA, Vugts HF, Rawaqa TT 1999 Evaporation from *Pinus caribaea* plantations on former grassland soils under maritime tropical conditions. Water Resources Research 35:2133–2144. 10.1029/1999WR900006

[PLV043C161] Watts DA, Moore GW 2011 Water-use dynamics of an invasive reed, *Arundo donax*, from leaf to stand. Wetlands 31:725–734. 10.1007/s13157-011-0188-1

[PLV043C162] West GB, Brown JH, Enquist BJ 1999 A general model for the structure and allometry of plant vascular systems. Nature 400:664–667. 10.1038/23251

[PLV043C163] White E, Vivian-Smith G, Barnes A 2009 Variation in exotic and native seed arrival and recruitment of bird dispersed species in subtropical forest restoration and regrowth. Plant Ecology 204:231–246. 10.1007/s11258-009-9587-2

[PLV043C164] Wicht CL 1945 Report of the committee on the preservation of the vegetation of the South Western Cape. Cape Town, South Africa: Special Publication of the Royal Society of South Africa.

[PLV043C165] Wilcox BP, Thurow TL 2006 Emerging issues in rangeland ecohydrology: vegetation change and the water cycle. Rangeland Ecology & Management 59:220–224. 10.2111/05-090R1.1

[PLV043C166] Wilcox BP, Huang Y, Walker JW 2008 Long-term trends in streamflow from semiarid rangelands: uncovering drivers of change. Global Change Biology 14:1676–1689. 10.1111/j.1365-2486.2008.01578.x

[PLV043C167] Williams CG, LaDeau SL, Oren R, Katul GG 2006 Modeling seed dispersal distances: implications for transgenic *Pinus taeda*. Ecological Applications 16:117–124. 10.1890/04-1901 16705965

[PLV043C168] Wise RM, Dye PJ, Gush MB 2011 A comparison of the biophysical and economic water-use efficiencies of indigenous and introduced forests in South Africa. Forest Ecology and Management 262:906–915. 10.1016/j.foreco.2011.05.021

[PLV043C169] Wise RM, van Wilgen BW, Le Maitre DC 2012 Costs, benefits and management options for an invasive alien tree species: the case of mesquite in the Northern Cape, South Africa. Journal of Arid Environments 84:80–90. 10.1016/j.jaridenv.2012.03.001

[PLV043C170] Woodward FI, Lomas MR 2004 Vegetation dynamics—simulating responses to climatic change. Biological Reviews 79:643–670. 10.1017/S1464793103006419 15366766

[PLV043C171] Wright IJ, Reich PB, Westoby M 2001 Strategy shifts in leaf physiology, structure and nutrient content between species of high- and low-rainfall and high- and low-nutrient habitats. Functional Ecology 15:423–434. 10.1046/j.0269-8463.2001.00542.x

[PLV043C172] Zavaleta E 2000 Valuing ecosystem services lost to Tamarix invasion in the United States. In: Mooney HA, Hobbs RJ, eds. Invasive species in a changing world. Washington, DC: Island Press, 261–300.

[PLV043C173] Zeppel M 2013 Convergence of tree water use and hydraulic architecture in water-limited regions: a review and synthesis. Ecohydrology 6:889–900.

[PLV043C175] Zhang L, Dawes WR, Walker GR 1999 Predicting the effect of vegetation changes on catchment average water balance. Technical Report 99/12, Cooperative Centre for Catchment Hydrology Australia: CSIRO.

[PLV043C176] Zhang L, Dawes WR, Walker GR 2001 Response of mean annual evapotranspiration to vegetation changes at catchment scale. Water Resources Research 37:701–708. 10.1029/2000WR900325

[PLV043C177] Zhang L, Potter N, Hickel K, Zhang Y, Shao Q 2008 Water balance modeling over variable time scales based on the Budyko framework—Model development and testing. Journal of Hydrology 360:117–131. 10.1016/j.jhydrol.2008.07.021

[PLV043C174] Zhang YQ, Chiew FHS 2012 Estimation of mean annual runoff across southeast Australia by incorporating vegetation types into Budyko-framework. Australian Journal of Water Resources 15:1–12. 10.7158/W10-840.2012.15.2

[PLV043C178] Zhao F, Xu Z, Zhang L 2012 Changes in streamflow regime following vegetation changes from paired catchments. Hydrological Processes 26:1561–1573. 10.1002/hyp.8266

